# Gender differences in transdiagnostic domains and function of adults measured by DSM-5 assessment scales at the first clinical visit: a cohort study

**DOI:** 10.1186/s12888-023-05207-8

**Published:** 2023-10-02

**Authors:** Erika F.H. Saunders, Megan Brady, Dahlia Mukherjee, Ritika Baweja, Lauren N. Forrest, Hassaan Gomaa, Dara Babinski, Fan He, Amanda M. Pearl, Duanping Liao, Daniel A. Waschbusch

**Affiliations:** 1 Department of Psychiatry and Behavioral Health, Penn State College of Medicine and Penn State Health Milton S. Hershey Medical Center, 500 University Drive, Hershey, PA 17033 USA; 2 Department of Psychiatry, Perelman School of Medicine, University of Pennsylvania, Philadelphia, PA USA; 3 Department of Public Health Sciences, Penn State College of Medicine, Hershey, PA USA

**Keywords:** Measurement-based care, Transdiagnostic, Functioning

## Abstract

**Background:**

Measurement-based care has been called for as best practice in psychiatric care and learning health systems and use of transdiagnostic measures was suggested as part of the DSM-5. Our objective is to examine gender differences in first visit socioeconomic, transdiagnostic, and functional characteristics of a dynamic, real-world measurement-based care cohort.

**Methods:**

Transdiagnostic, functional, and clinical measures were collected from 3,556 patients at first visit in an ambulatory psychiatric clinic. All patients were evaluated at the first visit by board-certified psychiatrists or licensed clinical psychologists. Demographic variables and clinical diagnoses were collected from the Electronic Medical Record. Self-report measures were collected that assessed transdiagnostic symptoms (DSM-5 Level 1 Cross-cutting Measure and Level 2 symptom scales), disability, alcohol use, attention deficit hyperactivity disorder (ADHD) symptoms, depression, anxiety, mania, suicidal thoughts and behaviors, and trauma exposure.

**Results:**

Men and women did not differ in age, BMI, household income, high school graduation rate, race, or ethnicity, but women were more likely to be formerly married and less likely to have commercial insurance. Compared to men, women reported significantly higher overall psychopathology on the transdiagnostic Level 1 Cross-cutting measure and had higher depression, anxiety, sleep, anger, ADHD combined presentation, and suicidality severity. Women also had higher disability scores than men. However, men reported higher alcohol, tobacco and substance use, and more risky behavior than women. Trauma exposure differed significantly by gender; men reported more exposure to accidents, war-related trauma, serious accidents, and major disasters and women reported more unwanted sexual contact.

**Conclusions:**

This cross-sectional study of a transdiagnostic, ecologically-valid real-word measurement-based care cohort demonstrates gender differences in socioeconomic factors, trauma exposure, transdiagnostic symptoms, and functioning.

**Supplementary Information:**

The online version contains supplementary material available at 10.1186/s12888-023-05207-8.

## Introduction

Psychiatric disorders are the second leading cause of disability in the world [1], and despite expansion of evidence-based psychotherapeutic and pharmacological options, public health measures of mental health indicate that the suicide rate has risen by 33% in the past two decades and the rates of deaths of despair have risen dramatically [2, 3]. These trends have worsened in the past year, given the COVID-19 pandemic and the resulting public health and socioeconomic consequences: In June 2020, 40% of the US adult population reported at least one symptom of adverse mental or behavioral health condition [4]. Improved interventions are desperately needed for our patients and our society.

One way to improve psychiatric interventions is through standardized assessments and measurement-based care [5–7], as clinics that implement these systems consistently show that their patients experience better outcomes compared to usual care [8–10]. Measurement-based care (MBC) has been defined as the use of systematic data on clinical outcomes collected at every patient visit to guide care [11–13]. MBC is critical to the National Academies of Medicine’s proposal to create a learning healthcare system, in which care can be iteratively improved through measurement and quality improvement cycles (14). A barrier to implementing MBC in mental health clinics is that patients present with multiple problems reflecting complex diagnostic challenges, and traditionally, symptom assessments are administered based on a patient’s presenting problem/diagnosis alone.

A contrast to the traditional approach of measuring symptoms in one domain based on a single index problem are several conceptual transdiagnostic frameworks proposed in recent years that recognize the need to account for high comorbidity amongst psychiatric and psychological syndromes when discussing etiological, phenomenological and clinical factors [15, 16]. The Research Domain Criteria (RDoC) framework [17, 18] identifies transdiagnostic domains by which to study etiological and risk factors of clinical and behavioral manifestations of psychopathology. This framework proposes that psychiatric disorders may be best understood through a dimensional framework, where underlying units of analysis (genes, molecules, cells, circuits, physiology, paradigm, behavior, self-report) can be used across six domains (negative valance systems, positive valance systems, cognitive systems, systems for social processes, arousal and regulatory systems, sensimotor systems) to understand underlying factors that lead to common clinical psychopathology.

An alternate transdiagnostic framework has been developed in the Hierarchical taxonomy of Psychopathology (HiTOP), a proposed system of hierarchical dimensional classification of mental and behavioral illness [19]. The HiTOP model conceptualizes psychopathology dimensionally, through a data-driven approach to construct syndromes based on covariation of symptoms divided into levels based on sign/symptom components and maladaptive traits, syndromes, subfactors (clusters of strongly related syndromes), spectra (broad groups of subfactors), and a general psychopathology factor reflecting overall maladaptation [15, 19, 20]. Ruggero and colleagues have proposed that HiTOP may be a useful clinical tool to communicate case conceptualization and treatment planning, while recognizing there are currently some practical limitations to implementation [15]. While RDoC and HiTOP have been posited as alternatives to the widely used clinical classification system of the Diagnostic and Statistical Manual of Mental Disorders (DSM) and International Classification of Disorders (ICD), the fifth version of the Diagnostic and Statistical Manual of Mental Disorders (DSM-5) introduced a dimensional framework and compilation of transdiagnostic assessments to screen and follow problems over time, the Level 1 Cross-Cutting Symptom Measure and Level 2 self-report measures [21, 22].

Gender differences have not been reported for the DSM-5 Level 1 Cross-Cutting Symptom Measure [21, 22]. However, consideration of gender in discussion of transdiagnostic domains has great value for the field and aligns with National Institutes of Health (NIH) priorities. For example, in 2016 the NIH highlighted the need to examine sex as a key variable in biological research studies [23, 24]. Moreover, biological sex and self-identified gender play a large role in many brain-based disorders and the psychological and social processes that influence risk and outcome for people seeking help in mental health clinics [25, 26].

To this end, we report on gender differences within a transdiagnostic framework in the first adult cohort from a real-world, naturalistic clinical sample, which we call the Penn State Psychiatry Clinical Assessment and Rating System (PCARES) Registry. This registry houses patient-reported assessments (using DSM-5 Assessment Measures and clinical data) gathered during routine clinical care of patients seeking mental health care at a mid-Atlantic clinic. Our goal in this report is to examine the use of existing evidence-based assessment tools to help clinicians elicit symptoms, behaviors, socioeconomic factors, trauma exposure and life functioning regardless of diagnostic categorization. In this report, we analyze gender differences in socioeconomic factors, trauma exposure, transdiagnostic domains, diagnosis-specific measures, and functional characteristics reported at patients’ first clinic visits.

## Methods

### Participants

We retrospectively analyzed data from 3,556 patients from the PCARES Registry, enrolled from 2/17/2015 to 5/30/2020. This sample is comprised of initial patient encounters (first visit) from a single mental health clinic in the mid-Atlantic region, which includes child services, general adult, and specialty services in Mood Disorders, Geriatrics, and Attention-Deficit/Hyperactivity Disorder (ADHD). Results from the child services program were reported by Waschbusch and colleagues [27]. The PCARES registry itself was considered a clinical quality improvement project by the relevant Institutional Review Board (IRB). It did not meet the criteria for human subject research, therefore IRB approval was not required and the need for consent to participate was waived. However, any research project using the de-identified PCARES data, including this project, requires approval by the relevant IRB. The current study was conducted according to the ethical principles of the Declaration of Helsinki and approved by the Pennsylvania State University IRB (IRB #183 and #7926). All patients were evaluated by board-certified psychiatrists or licensed clinical psychologists. All patients were included in the registry regardless of psychiatric diagnosis or purpose of visit. The PCARES Registry includes self-report measures and select diagnostic and demographic information extracted from patients’ electronic medical records (EMR). Although previous studies using these data have been published (28–31), the current study’s aims are unique and have never been reported.

### Demographics

Demographic data were extracted from the EMR. Demographic data included self-identified gender (male, female, patient declined, unavailable), race (American Indian/Alaskan Native, Asian, Black or African American, Native Hawaiian or Pacific Islander, White, two or more races, other race, patient declined, unavailable), ethnicity (Hispanic, Latino, or Spanish origin; not Hispanic, Latino, or Spanish origin; multiple; patient declined; unavailable), and marital status (single, married, and formerly married including divorced, separated, and widowed, patient declined, unavailable). Two steps were used to create participants’ socioeconomic profiles. First, participants’ home address/zip code were extracted from the EMR. Second, education and income at the zip code-level were obtained from the 2016 American Community Survey (ACS) five-year estimates database (32) so that we could identify the education and median income for each patient’s zip code. We also obtained the county level rural/urban profile from the Center for Rural PA, and classified patients into binary categories by county (rural/urban) and municipality (rural/urban). Insurance status was classified as follows: commercial insurance included preferred provider organizations (PPO), Blue Cross/Blue Shield related organizations, health maintenance organizations (HMO), and other commercial insurance payers (state-funded Medicaid payers, Medicare, or self-pay).

### Clinical diagnoses

Clinical diagnoses were extracted from the first visit EMR documentation. Body mass index (BMI) was calculated from measured height and weight recorded in the EMR during the first visit.

### Self-report measures

Participants completed a battery of self-report measures at the first visit. However, the battery was changed based on clinician feedback midway through data collection. Herein, we report first-visit data from the first and second versions of the battery, termed “battery 1” and “battery 2” (see Supplemental Table 1 for details of battery schedule). Self-report measures were available in the English language.

### Trauma exposure

The Brief Trauma Questionnaire (BTQ) was used to report lifetime trauma exposure [33].

### Transdiagnostic symptom measures

The DSM-5 Level 1 Cross-Cutting Symptom Measure [21, 22] is a self-report screening tool assessing depression, mania, anxiety, somatic symptoms, suicidal ideation, psychosis, sleep, memory, obsessions and compulsions, dissociation, personality styles, and substance use. If participants reported that anger, anxiety, depression, mania, obsessions and compulsions, sleep, or somatic symptoms bothered them more than slightly or rarely, the screener was determined to be positive. In battery 1, if the Level 1 screener was positive, the patient completed the Level 2 in-depth questionnaire for that symptom area. The DSM-5 Level 2 adult assessments included the PROMIS Emotional Distress, Depression Short Form; PROMIS Emotional Distress, Anger Short Form; PROMIS Emotional Distress, Anxiety Short Form; and PROMIS, Sleep Disturbance Short Form. Each of these Level 2 assessments was scored using T scores. The Personality Inventory for DSM-5-Brief Form (PID-5-BF) was used to assess personality traits and domains. The Columbia Suicidality Severity Rating Scale (CSSRS) was used to assess suicide risk [34].

In battery 2, regardless of whether patients screened positive for any Level 1 items, all patients completed the following measures. The Patient Health Questionnaire (PHQ-9) assessed depressive symptoms [35]. The Generalized Anxiety Disorder scale (GAD-7) assessed anxiety [36]. The Altman Self-Rating Mania Scale (ASRM) assessed manic symptoms [37]. The Mood Disorder Questionnaire (MDQ) screened for symptoms of bipolar disorder [38]. The Adult ADHD self-report scale (ASRS) [39] assessed for any ADHD, inattentive presentation, hyperactive presentation, or combined presentation.

### Risky behavior

The Alcohol Use Disorders Identification Test (AUDIT) [40] screened for risky alcohol or substance use.

### Functioning

The WHO Disability Assessment Schedule 2.0 categorized disabilities in cognition, mobility, self-care, getting along with others, ability to participate in life activities such as household and school or work activities, and social participation. Overall summary score and domain scores were calculated by using the item-response-theory based scoring method [41].

### Statistical analysis

To compare the demographic and clinical characteristics between men and women, t-tests and chi-square (or Cochran-Mantel–Haenszel) tests were used for continuous and categorical variables, as appropriate. To enhance the interpretability of the comparisons, we further calculated the effect sizes for the gender differences for (1) continuous variables as Cohen’s d [i.e., (Mean_women_ – Mean_men_/SD_overall_)]; and (2) categorical variables as difference in proportions (i.e., %_women_ - %_men_). SAS 9.4 was used to perform all statistical analyses. Statistical significance was set at *p* ≤ 0.05.

## Results

### Demographic characteristics

This PCARES Registry cohort included 3,556 individuals (37% men, 63% women) with a mean age of 42.2 ± 17.0 years (see Table 1). Most participants (84.5%) identified as White; 5.9% identified as Black or African American, 9.7% identified as another race, and 4.4% identified as Hispanic. Participants’ marital status included 47% who reported being single, 37% married, and 16% formerly married. Almost half the sample included participants who reported having public insurance or self-paying for healthcare. Demographic factors of age, education (mean high school graduation rate 90.1%, SD 4.6%), and race and ethnicity did not differ significantly between men and women. Household median income (mean $59,815, SD $10,973) did not differ between men and women, and was below the median household income for Pennsylvania 2015–2019 [42]. Significant differences by gender were reported in marital status (more women [19%] than men [11%] were formerly married) and insurance type (more men [59%] than women [55%] with commercial insurance). Rurality distribution included 5.2% in rural county/rural municipality (N = 180); 2.4% in rural county/urban municipality (N = 85); 8.8% in urban county/rural municipality; and 83.6% urban county/urban municipality (N = 2923). BMI did not differ by gender.

**Table 1:** Patient demographics and trauma exposure stratified by gender

Variable	Overall		Men		Women		P
	N = 3556		N = 1305		N = 2251		
	Mean	SD	Mean	SD	Mean	SD	
**Age (year)**	42.4	17.0	41.8	17.2	42.7	16.8	0.15
**BMI (kg/m** ^**2**^ **)**	30.3	8.5	30.0	8.3	30.6	8.5	0.07
**Household median income ($)**	59,815	10,973	60,018	11,198	59,698	10,841	0.40
**≥High school graduates (%)**	90.1	4.6	90.1	4.8	90.2	4.4	0.47
	**%**	**N**	**%**	**N**	**%**	**N**	
**Race**							
White	84.5	3004	85.7	1118	83.8	1886	
Black or African American	5.9	208	5.6	73	6.0	135	0.29
Other	9.7	344	8.7	114	10.2	230	
**Ethnicity**							
Hispanic	4.4	156	4.1	53	4.6	103	
Not Hispanic Other	91.8 4.8	3264 136	91.8 4.1	1198 54	91.8 3.6	2066 82	0.60
**Marital status**							
Single	47.3	1594	50.0	652	41.9	942	
Married	37.2	1383	38.9	508	38.9	875	**< 0.01**
Formerly Married	15.5	579	11.1	145	19.3	434	
**Primary insurance type**							
Commercial	56.7	2009	59.2	769	55.2	1240	**0.02**
Public/Self-Pay	43.3	1536	40.8	531	44.8	1005	
**Secondary insurance type**							
Commercial	13.6	474	12.6	162	14.2	312	0.20
Public/Self-Pay	86.4	3017	87.4	1124	85.8	1893	
**Trauma (BTQ)**	**Overall N = 1117**		**Men N = 356**		**Women N = 761**		**P**
	**%**	**N**	**%**	**N**	**%**	**N**	
War Zone, Casualty	2.5	28	6.5	23	0.7	5	**< 0.01**
Serious Accident	27.1	296	31.8	111	24.9	185	**0.02**
Major Disaster	15.0	166	19.8	70	12.8	96	**< 0.01**
Life-threating Illness	16.2	177	18.9	66	14.9	111	0.10
Child Abuse	26.8	295	25.1	88	27.6	207	0.37
Physical Attack	27.8	304	27.8	97	27.8	207	0.99
Unwanted Sex	34.5	378	13.6	48	44.4	330	**< 0.01**
Seriously Injured	21.9	239	23.6	83	21.1	156	0.35
Others Died Violently	20.2	223	22.7	80	19.0	143	0.16
Others Injured Seriously	24.3	261	29.3	101	21.9	160	**< 0.01**
Patients with > = 1 “Yes”							
	**Mean**	**SD**	**Mean**	**SD**	**Mean**	**SD**	
Number of “Yes”	2.1	2.0	2.2	2.0	2.1	1.9	0.68

BTQ = Brief Trauma Questionnaire

### Trauma

Men reported higher frequency of exposure to war-related trauma, serious accidents, major disasters, and seeing others injured seriously categories than women. Women reported significantly higher frequency of unwanted sex than men (Table 1).

### Clinical Diagnosis

 The most common diagnoses were major depressive disorder (41.2%), bipolar disorder (10.4%), and generalized anxiety disorder (21.5%; see Table 2). In order of the magnitude of gender difference by effect size (ES), more women were diagnosed with major depressive disorder (MDD) (44.1%, ES = 8.3% ), generalized anxiety disorder (22.9%, ES = 4.0%), post-traumatic stress disorder (PTSD) (7.7%, ES = 3.8%), panic disorder (6%, ES = 2.1%), bipolar II disorder (3.7%, ES = 1.7%), and eating disorders (1.4%, ES = 1.2%), while more men were diagnosed with autism spectrum disorder (10.9%, ES=-8.4%), ADHD (9.8%, ES=-4.5%), and psychotic disorder (4.5%, ES=-2.0%). Women had more diagnoses than men, with a mean of 1.6 compared to 1.4 for men, with a small effect size (Cohen’s d = 0.18, *p* < 0.01).

**Table 2:** Clinical Diagnoses stratified by gender

Variable	Overall N = 3556		Men N = 1305		Women N = 2251		Effect size^†^	P
	%	N	%	N	%	N		
MDD-Lifetime	41.2	1461	36.1	470	44.1	991	8.3%	**< 0.01**
MDD-Current	37.8	1341	32.3	420	41.0	921	8.7%	**< 0.01**
Any Bipolar-Lifetime	10.4	368	9.2	119	11.1	249	1.9%	0.07
Bipolar I-Lifetime	3.0	106	3.0	39	3.0	67	0.0%	0.98
Bipolar II-Lifetime	3.0	108	2.0	26	3.7	82	1.7%	**< 0.01**
Bipolar NOS-Lifetime	4.6	162	4.3	56	4.7	106	0.4%	0.57
Generalized Anxiety Disorder	21.5	761	18.9	246	22.9	515	4.0%	**< 0.01**
Panic Disorder	5.2	185	3.9	51	6.0	134	2.1%	**< 0.01**
Social Phobia	2.4	85	2.5	32	2.4	53	-0.1%	0.85
Psychotic Disorder-Lifetime	3.2	114	4.5	59	2.5	55	-2.0%	**< 0.01**
Obsessive Compulsive Disorder	3.0	108	3.3	43	2.9	65	-0.4%	0.49
Post-traumatic Stress Disorder	6.3	224	3.9	51	7.7	173	3.8%	**< 0.01**
Antisocial Personality Disorder	0.1	2	0.2	2	0.0	0	-0.2%	N/A
Eating Disorders	1.0	35	0.2	3	1.4	32	1.2%	**< 0.01**
Alcohol Use Disorders	1.6	58	2.2	29	1.3	29	-0.9%	**0.03**
Substance Use Disorders	1.6	56	2.0	26	1.3	30	-0.7%	0.13
Opioid Use Disorder	0.3	10	0.3	4	0.3	6	0.0%	0.99
Somatic Disorders	0.7	24	0.7	9	0.7	15	0.0%	0.93
Other Personality Disorders	1.2	43	0.8	10	1.5	33	0.7%	0.07
Autism Spectrum Disorder	5.6	198	10.9	142	2.5	56	-8.4%	**< 0.01**
ADHD	7.0	248	9.8	128	5.3	120	-4.5%	**< 0.01**
	**Mean**	**SD**	**Mean**	**SD**	**Mean**	**SD**		
# of diagnoses^††^	1.5	1.1	1.4	1.0	1.6	1.1	0.18	**< 0.01**

MDD = Major Depressive Disorder

NOS = Not Otherwise Specified

ADHD = Attention Deficit Hyperactivity Disorder

†: Effect size for categorical variable = % (women) - % (men);

Effect size for continuous variable = [Mean (women) – Mean (men)]/SD (overall)

††: # of diagnoses was calculated as the sum of all clinical diagnoses listed above, except “Any Bipolar-Lifetime”

### Transdiagnostic measures

 The percentage of positive DSM-5 Level 1 Cross-Cutting Symptom Measure screener areas (Table 3) differed between men and women for most items. In order of the magnitude of difference by ES, women had higher rates of anxiety (ES = 8.4%), somatic symptoms (ES = 7.2%), anger (ES = 7.1%), dissociation (ES = 7.0%), memory (ES = 5.6%), depression (ES = 4.5%), personality symptom domains (ES = 4.3%), and sleep disturbance (ES = 4.2%), whereas men had higher rates of tobacco use (ES=-4.7%), psychosis (ES=-3.3%), and substance use (alcohol and other substances (ES=-2.4%). Women reported significantly higher DSM Level 1 Cross-Cutting Measure overall symptom score and number of symptom domains than men, with small effect sizes. In the DSM-5 Level II symptom scores, women had significantly higher mean T-scores in sleep, depression, anxiety, and anger than men. Similarly, on the battery 2 measures (Table 4), women had higher scores on depression (PHQ-9, ES = 0.30) and anxiety (GAD-7, ES = 0.31), and higher rates of ADHD combined presentation, as compared to men (ASRS, ES = 8.1%). On the MDQ, men scored higher than women on the risky behavior item (ES=-7%). On the PID-5-BF, women reported significantly higher negative affect and detachment, while men reported higher antagonism and disinhibition (Table 3). Women scored higher than men on suicidality (ES = 0.14, Table 3).

**Table 3:** Transdiagnostic measure, personality, suicide and alcohol/substance use screeners stratified by gender

DSM-5 Level I^†^	Overall N = 2350		Men N = 785				Women N = 1565			Effect size^*^	P
	%	N	%		N		%		N		
Depression	81.5	1913	78.5		614		83.1		1299	4.6%	**< 0.01**
Anger	69.6	1630	64.9		507		72.0		1123	7.1%	**< 0.01**
Mania	42.1	976	42.4		327		42.0		649	-0.4%	0.87
Anxiety	83.1	1943	77.5		605		85.9		1338	8.4%	**< 0.01**
Somatic	69.9	1626	65.1		506		72.3		1120	7.2%	**< 0.01**
Suicide	29.5	688	28.1		219		30.2		469	2.1%	0.30
Psychosis	14.8	345	17.0		132		13.7		213	-3.3%	**0.04**
Sleep Disturbance	67.7	1583	64.9		506		69.1		1077	4.2%	**0.04**
Memory	45.0	1049	41.2		322		46.8		727	5.6%	**0.01**
OCD	45.9	1063	47.4		366		45.2		697	-2.2%	0.31
Dissociation	35.3	819	30.6		236		37.6		583	7.0%	**< 0.01**
Personality	64.6	1501	61.8		478		66.1		1023	4.3%	**0.04**
Alcohol Use	5.0	115	7.5		58		3.7		57	-3.8%	**< 0.01**
Tobacco Use	22.0	511	25.1		195		20.4		316	-4.7%	**< 0.01**
Substance Use	6.6	152	8.2		63		5.8		89	-2.4%	**0.03**
	**Mean**	**SD**	**Mean**		**SD**		**Mean**		**SD**		
Summary score	30.5	16.4	28.6		16.9		31.4		16.0	0.17	**< 0.01**
Symptom domains, #	6.6	3.2	6.3		3.3		6.7		3.1	0.13	**< 0.01**
**DSM-5 Level II** ^**††**^	**Overall N = 1253**		**Men N = 412**				**Women N = 841**				**P**
	**Mean**	**SD**	**Mean**		**SD**		**Mean**		**SD**		
Depression T-score	65.1	8.7	63.5		8.7		65.8		8.7	0.26	**< 0.01**
Anxiety T-score	65.9	8.8	64.5		8.8		66.5		8.7	0.23	**< 0.01**
Sleep T-score	62.9	7.9	61.2		7.4		63.7		8.0	0.32	**< 0.01**
Anger T-score	62.8	10.5	61.6		10.2		63.3		10.6	0.16	**0.02**
**PID-5-BF** ^**††**^	**Overall N = 1372**		**Men N = 465**				**Women N = 906**				**P**
	**Mean**	**SD**	**Mean**		**SD**		**Mean**		**SD**		
Negative Affect	1.5	0.9	1.4		0.8		1.6		0.9	0.22	**< 0.01**
Detachment	1.1	0.9	1.1		0.9		1.2		0.9	0.11	**< 0.01**
Antagonism	0.3	0.6	0.4		0.6		0.3		0.5	-0.17	**< 0.01**
Disinhibition	0.8	0.8	0.9		0.8		0.8		0.8	-0.13	**0.05**
Psychoticism	0.7	0.8	0.7		0.8		0.7		0.8	0.0	0.14
**Suicide (C-SSRS** ^**†††**^ **)**	**Overall N = 2623**		**Men N = 956**			**Women N = 1667**					**P**
	**%**	**N**	**%**	**N**		**%**		**N**			
None	69.8	1830	73.2	700		67.9		1153		-5.3%	
Wished you were dead	10.2	268	8.4	80		11.3		188		2.9%	
Think of killing yourself	3.4	89	4.0	38		3.1		53		-0.9%	
How may kill yourself	3.7	98	4.3	41		3.4		57		-0.9%	**< 0.01**
Intention of act	1.1	30	1.4	13		1.0		17		-0.4%	
Work out details	0.7	19	0.7	7		0.7		13		0.0%	
Attempts/harm yourself	6.7	175	5.1	49		7.6		126		2.5%	
“Yes” within 3 months	7.4	13	12.2	6		5.6		7		-6.6%	0.13
Start to kill yourself	4.4	114	2.9	28		5.2		87		2.3%	
“Yes” within 3 months	27.2	31	35.7	10		24.4		21		-11.3%	0.25
	**Mean**	**SD**	**Mean**	**SD**		**Mean**		**SD**			
Continuous Score	1.1	2.1	0.9	1.9		1.2		2.2		0.14	**< 0.01**
**AUDIT**	**Overall N = 1119**		**Men N = 358**			**Women N = 761**					**P**
	**Mean**	**SD**	**Mean**	**SD**		**Mean**		**SD**			
Alcohol Use	3.0	4.2	3.5	4.5		2.8		4.0		-0.07	**0.01**
Substance Use	1.5	2.6	1.8	3.0		1.3		2.4		-0.19	**< 0.01**

AUDIT = Alcohol Use Disorders Identification Test

^†^ DSM-5 Level 1 and 2 Cross-Cutting Symptom Measure screeners scoring algorithm in Supplemental Table 2

^††^: DSM-5 Level 1 and 2 Cross-Cutting Symptom Measure screeners and Personality Inventory for DSM-5 Brief Form **(**PID-5-BF) scores were only available from cohort 1

††: Columbia-Suicide Severity Rating Scale (C-SSRS) score equals to the question number of the “highest” (i.e. most severe suicidal thoughts or behaviors) question with “Yes” as the answer. The column percentages/Ns are mutually exclusive

The prevalence of “Yes within 3 months” for Q6 and Q7 were calculated within those who scored 6 or 7, respectively. For example, among the 175 patients who scored “6” (“Yes” on attempted to kill or harm yourself), 7.4% (n = 13) of them took actions within the last 3 month

*: Effect size for categorical variable = % (women) - % (men);

Effect size for continuous variable = [Mean (women) – Mean (men)]/SD (overall)

**Table 4:** Mania, Depression, Anxiety, ADHD, Bipolar Disorder measures stratified by gender

Mania (ASRM)	Overall N = 2564		Men N = 931		Women N = 1633		Effect size*	P
	Mean	SD	Mean	SD	Mean	SD		
Summary Score	4.48	4.25	4.57	4.27	4.43	4.23	-0.03	0.42
**Depression (PHQ-9)**	**Overall N = 2797**		**Men N = 1026**		**Women N = 1771**			**P**
	**Mean**	**SD**	**Mean**	**SD**	**Mean**	**SD**		
Summary Score	10.7	7.1	9.4	7.1	11.5	7.0	0.30	**< 0.01**
**Anxiety (GAD-7)**	**Overall N = 2624**		**Men N = 964**		**Women N = 1660**			**P**
	**Mean**	**SD**	**Mean**	**SD**	**Mean**	**SD**		
Summary Score	9.5	6.5	8.2	6.4	10.2	6.4	0.31	**< 0.01**
**Attention Deficit Disorders (ASRS** ^**†**^ **)**	**Over N = 940**		**Men N = 314**		**Women N = 626**			**P**
	**%**	**N**	**%**	**N**	**%**	**N**		
Any ADHD	54.7	514	51.0	160	56.6	354	5.6%	0.10
Inattentive Presentation	24.8	233	25.2	79	24.6	154	-0.6%	0.85
Hyperactive Presentation	5.4	51	6.7	21	4.8	30	-1.9%	0.23
Combined Presentation	24.5	230	19.1	60	27.2	170	8.1%	**< 0.01**
**Bipolar Disorders (MDQ)**	**Overall N = 1068**		**Men N = 343**		**Women N = 725**			**P**
	**%**	**N**	**%**	**N**	**%**	**N**		
Feel Good/Hyper	25.9	274	28.2	96	24.8	178	-3.4%	0.24
More Self-confident	26.6	283	29.1	99	25.4	184	-3.7%	0.20
Less sleep	27.2	289	29.8	102	26.0	187	-3.8%	0.19
More Talkative	34.3	364	33.2	113	34.8	251	1.6%	0.61
Can’t Slow Mind Down	65.4	691	66.5	224	64.9	467	-1.6%	0.61
Easily Distracted	68.0	721	64.5	218	69.7	504	5.2%	0.09
More Energy	27.9	296	29.0	99	27.4	197	-1.6%	0.58
More Active	29.3	306	29.9	100	29.0	206	-0.9%	0.78
More Social	14.9	158	17.0	58	13.9	100	-3.1%	0.18
More Interested in Sex	23.1	244	24.9	84	22.2	160	-2.7%	0.33
Risky Behaviors	21.9	231	26.6	90	19.6	141	-7.0%	**0.01**
Spending More Money	20.5	217	19.3	65	21.1	152	2.8%	0.49
Possible Bipolar^†††^	18.3	166	19.0	55	18.0	111	-1.0%	0.71
	**Mean**	**SD**	**Mean**	**SD**	**Mean**	**SD**		
Number of “Yes”	4.1	3.7	4.2	3.8	4.0	3.7	-0.05	0.40

ASRM = Altman Mania Rating Scale

PHQ-9 = Patient Health Questionnaire – 9

GAD-7 = Generalized Anxiety Disorder − 7

ASRS = Adult ADHD Severity Rating Scale

MDQ = Mood Disorder Questionnaire (MDQ)

†: ASRS was only available from patients in Cohort 1. Positive symptom endorsement on the ASRS was based on procedures outlined by Kessler and colleagues [39]

**ADHD inattentive presentation**: having at least 5 or more symptoms of inattention and less than 5 symptoms of hyperactivity/impulsivity

**ADHD hyperactive presentation**: having at least 5 or more symptoms of hyperactivity/impulsivity and less than 5 symptoms of inattention

**ADHD combined presentation**: having at least 5 or more symptoms of inattention AND 5 or more symptoms of hyperactivity/impulsivity

†††: Possible Bipolar in the MDQ is defined as (1) Having ≥ 7 manic symptoms; and (2) Multiple of them occurred as the same time; and (3) These symptoms had caused moderate or serious problems in their lives

*: Effect size for categorical variable = % (women) - % (men);

Effect size for continuous variable = [Mean (women) – Mean (men)]/SD (overall)

### Substance use

Men scored significantly higher than women on the AUDIT alcohol (ES=-0.07) and substance use subscales (ES=-0.19, Table 3).

### Functioning

Disability scores (Table 5) indicated that the sample overall had worse functioning than 88% of the general population [43]. Women reported significantly greater disabilities in all domains compared to men, with disparities most apparent in life activities (ES = 0.24), mobility (ES = 0.19) and social participation (ES = 0.19). Both women and men rated social participation as the most impaired domain and self-care as the least impaired domain.

**Table 5:** Functioning as assessed by the World Health Organization Disability Assessment Scale (WHODAS) scores stratified by gender

WHODAS	Overall N = 3381		Men N = 1230		Women N = 2151		Effect Size^*^	P
	Mean	SD	Mean	SD	Mean	SD		
Cognition	29.6	24.5	27.3	24.1	30.9	24.6	0.15	**< 0.01**
Mobility	28.2	31.0	24.3	30.1	30.4	31.3	0.20	**< 0.01**
Self-care	14.1	22.5	12.5	21.7	15.1	22.8	0.12	**< 0.01**
Getting Along	34.8	28.9	33.4	29.7	35.5	29.9	0.07	**0.04**
Life Activities	47.4	28.1	42.8	27.6	49.6	28.1	0.24	**< 0.01**
Participation	46.0	28.2	42.6	28.1	47.9	28.1	0.19	**< 0.01**
Summary Score	31.9	21.1	28.9	20.7	33.5	21.2	0.22	**< 0.01**

*: Effect size = [Mean (women) – Mean (men)]/SD (overall)

## Discussion

We examined socioeconomic factors, trauma exposure, transdiagnostic measures, and functioning by gender at first visit in an ambulatory mental health clinic, including the first report to our knowledge of gender differences in the transdiagnostic instrument DSM-5 Level 1 Cross-Cutting Symptom Measure. Our sample was representative of the local population, and the gender balance of the sample is representative of broader treatment patterns seen for mental illness, with women seeking mental health care more often than men [44].

Women reported significantly higher severity of transdiagnostic psychopathology and number of comorbid mental illnesses, as well as a higher impact of mental health problems on functioning compared to men. This is contrary to other findings which indicate that men present with more severe mental illness at first visit, possibly as a result of increased reluctance to seek health care [45–47]. However, symptom prevalence on the DSM-5 Level I Cross-cutting Symptom Measure and the rates of diagnosis are consistent with previous findings that women have a higher rate of depressive disorders [48], bipolar II disorder [49], anxiety disorders [50], eating disorders [51], and PTSD [52]. We also found gender differences in trauma exposure, where women reported higher rates of unwanted sexual intercourse while men reported more experiences of war zone or casualty trauma. This generally maps onto known gender differences in lifetime trauma exposure [50].

While the diagnosis of ADHD was almost twice as high in men than in women, the combined presentation of ADHD self-report score was higher in women than men [53]. This is consistent with some research reporting that undiagnosed adults who screen positive for ADHD are more likely to be women versus men [54]. In a large meta-analysis, women with ADHD were more frequently diagnosed with the inattentive subtype, and men were diagnosed more frequently with combined subtype [55]. However, a study of the ASRS used in a sample of patients in mental health clinics found that gender differences were not noted in subtype scales [56]. One explanation for the difference is that self-report scales may pick up more subtle presentations that are not uncovered by clinicians. An alternate explanation is that clinicians are accounting for ADHD symptoms as a part of another disorder. Further work needs to be done to determine the benefit of screening instruments for ADHD in a clinical diagnostic approach.

Women had higher sleep disturbance than men as measured by DSM5 Level I and II screeners. While population-based studies demonstrate that women have higher percentage of sleep time and slow wave sleep, with less sleep disturbance in response to an external stressor than men [57], the predominance of mood and anxiety disorders in women could contribute to more women than men reporting sleep difficulties.

A greater proportion of women than men reported anger as a prominent symptom on the DSM5 Level 1 and Level II severity screeners. While subjective anger is not part of the diagnostic criteria for depression in adults and is not routinely measured in studies of mental illness that are not directly studying aggression or suicide, anger has been posited to be an “alternative” expression of low mood in depression. In fact, an interesting analysis of the National Comorbidity Study Replication (NCS-R) showed when anger and aggression were measured (along with risk taking and substance use) and scored as primary symptoms of depression, gender differences in diagnosis of depression disappeared [58, 59]. Because the NCS-R was a population-based sample and our sample is a naturalistic, clinic-based sample, the findings are not directly comparable. The finding of prominent anger in women highlights the importance of measuring symptoms in transdiagnostic domains in the clinic setting.

Somatic symptoms were more prevalent among women than men, consistent with available studies [60] [61]. Women also reported higher rates of suicidality than men, consistent with findings that women have higher rates of non-fatal suicide attempts and men have higher rates of suicide deaths [62]. Women reported higher negative affect and detachment, but lower antagonism and disinhibition than men in our sample, though effect sizes were small. While some studies show gender discrepancies in personality traits and disorders, others do not; the differences found here may be due to measuring personality traits through self-report compared to structured diagnosis [63]. While there may not be gender differences in population rates of personality traits or disorders, an interesting next question is how strongly expressed personality traits impact women and men in day-to-day functioning.

In our sample, women reported experiencing more impairment participating socially, accomplishing household tasks, and completing daily work or school activities compared to men. The greatest discrepancy between men and women was impairment in completing life activities. One potential explanation is that women are more commonly responsible for household tasks than men resulting in more opportunities for an impact in functioning (a floor effect in male functioning). Only a few studies have assessed gender differences in functional impairment associated with psychopathology [64]. Results similar to our study were reported from a large prospective multi-center study which found that mental health problems were more likely to affect women’s marital functioning but men’s work functioning [65]. Overall, the finding that psychiatric symptoms differentially impact functioning highlight the need to monitor in our patients both psychiatric symptoms themselves and how symptoms affect functioning.

## Strengths and Limitations

Strengths in our approach include using validated, transdiagnostic self-report measures in a framework recommended by the DSM-5 in a naturalistic, real-world cohort. These results have generalizability to patients seeking care in psychiatric clinics, however our sample includes a majority White, non-Hispanic, English speaking population and may not generalize to racial, ethnic and gender identity populations underrepresented in medical research. A limitation of using this approach is that data are gathered for clinical purposes and extracted from the EMR and the diagnostic description are not as comprehensive as structured interview [28]. The self-report measures do not adequately capture cognitive functioning and neurodevelopmental domains, and those with neurodevelopmental disorders experience more comorbid psychopathology. The sample is biased by the fact that they are presenting for clinical care and we cannot compare our sample directly to a group that did not obtain clinical care. We acknowledge the importance of gender expansive measurements, such as transgender or nonbinary identities, which have significant implications in mental health, however, the variable to represent gender is self-reported by patients during enrollment in the EMR and did not at the time allow for non-binary description of gender identity. Future studies should consider reporting gender expansive categories on these measures [66].

## Clinical implications

Questions remain in how to integrate such layered levels of data to personalize care. DSM-5 added the transdiagnostic assessment paradigm to promote the assessment of several dimensional areas across categorical diagnoses. Symptom scales that take a transdiagnostic domain approach and include social factors, trauma exposure and domains of functioning allow clinicians to more efficiently and accurately identify areas of concern and distress for patients. The impact of mental illness and psychopathology are affected by biological, psychological, social and cultural factors that influence both the presence of symptoms and resultant level of functioning.

We have found that the higher level of psychopathology and functional impairment, exposure to sexual trauma, and anger in women when compared to men at first treatment suggests that women are waiting longer in the course of illness to seek treatment than men. This may affect rates of recovery and highlights a need to promote earlier treatment intervention in women’s health. Because mental illnesses can have a severe impact on daily functioning, understanding the mediating and moderating factors between mental illness and impairment in men and in women may generate targets for further study, including data-driven approaches to treatment matching.

### Electronic supplementary material

Below is the link to the electronic supplementary material.

Supplementary Material 1 Supplemental Table 1: PCARES Battery

## Data Availability

The datasets used and/or analysed during the current study are available from the corresponding author on reasonable request.
