# Supplementary material for: Gender differences in transdiagnostic domains and function of adults measured by DSM-5 assessment scales at the first clinical visit: a cohort study
Source: BMC Psychiatry. 2023 Oct 2;23:709. doi: 10.1186/s12888-023-05207-8 (PMC10544467; doi:10.1186/s12888-023-05207-8)
Supplement: Supplementary file 1 — Supplementary Material 1 Supplemental Table 1: PCARES Battery [file 12888_2023_5207_MOESM1_ESM.docx]

Supplemental Table 1: PCARES Battery

| Measure | First Visit | Follow-up Visits |
| --- | --- | --- |
| Battery 1 |  |  |
| MINI Interview 6.0 | X | X |
| DSM-5 Level 1 Cross-Cutting Symptom Measure | X | X |
| DSM-5 Level 2 Scales | X | X |
| DSM-5 Severity Measures | X | X |
| Personality Inventory for DSM-5 – Brief Form | X |  |
| Alcohol Use Disorders Identification Test (AUDIT) | X |  |
| WHODAS 2.0 | X |  |
| Adult ADHD Self-Report Symptom Checklist | X |  |
|  |  |  |
| Battery 2 |  |  |
| DSM-5 Level 1 Cross-Cutting Symptom Measure | X |  |
| Alcohol Use Disorders Identification Test (AUDIT) | X |  |
| Brief Trauma Questionnaire | X |  |
| Mood Disorder Questionnaire | X |  |
| WHODAS 2.0 | X | X |
| Patient Health Questionnaire (PHQ-9) | X | X |
| Generalized Anxiety Disorder Scale (GAD-7) | X | X |
| Altman Self-Rating Mania Scale (ASRM) | X | X |
| Columbia-Suicide Severity Rating Scale | X | X |

MINI: Mini-International Neuropsychiatric Interview

WHODAS 2.0: World Health Organization Disability Assessment Scale 2.0

ADHD: Attention Deficit Hyperactivity Disorder

**Supplemental Table 2: DSM Level I Symptoms Algorithms**

| **Symptom Domain** | **Question numbers** | **Cutoff** |
| --- | --- | --- |
| Depression | Q1, Q2 | ≥ 2 |
| Anger | Q3 | ≥ 2 |
| Mania | Q4, Q5 | ≥ 2 |
| Anxiety | Q6, Q7, Q8 | ≥ 2 |
| Somatic | Q9, Q10 | ≥ 1 |
| Suicidal | Q11 | ≥ 1 |
| Psychotic | Q12, Q13 | ≥ 1 |
| Sleep disturbance | Q14 | ≥ 2 |
| Memory | Q15 | ≥ 2 |
| OCD | Q16, Q17 | ≥ 2 |
| Dissociation | Q18 | ≥ 2 |
| Personality | Q19, Q20 | ≥ 2 |
| Alcohol Use | Q21 | ≥ 2 |
| Tobacco Use | Q22 | ≥ 2 |
| Substance Use | Q23 | ≥ 2 |

A patient is considered as having the symptom if **any** of the question within the domain is above the cutoff. For example, a patient is considered as having level I depression, if either Q1 or Q2 was scored ≥ 2.
